# Phytochemical analysis and versatile in vitro evaluation of antimicrobial, cytotoxic and enzyme inhibition potential of different extracts of traditionally used *Aquilegia pubiflora* Wall. Ex Royle

**DOI:** 10.1186/s12906-021-03333-y

**Published:** 2021-06-07

**Authors:** Hasnain Jan, Hazrat Usman, Muzamil Shah, Gouhar Zaman, Sadaf Mushtaq, Samantha Drouet, Christophe Hano, Bilal Haider Abbasi

**Affiliations:** 1 Department of Biotechnology, Quaid-i-Azam University, Islamabad, 45320 Pakistan; 2 Laboratoire de Biologie des Ligneux et des Grandes Cultures (LBLGC EA1207), INRA USC1328, Plant Lignans Team, Université d’Orléans, Pôle Universitaire d’Eure et Loir, 21 rue de Loigny la Bataille, 28000 Chartres, France; 3 Bioactifs et Cosmétiques, GDR 3711 COSMACTIFS, CNRS/Université d’Orléans, 45067 Orléans, CÉDEX 2 France

**Keywords:** Anti-cancer, HPLC, Flavonoids, Antimicrobial, Anti-aging, Inflammatory

## Abstract

**Background:**

Himalayan Columbine (*Aquilegia pubiflora* Wall. Ex Royle) is a medicinal plant and have been used as traditional treatments for various human diseases including skin burns, jaundice, hepatitis, wound healing, cardiovascular and circulatory diseases. Till now there is no report available on phytochemical investigation of Himalayan Columbine and to the best of our knowledge, through present study we have reported for the first time, the phytochemical analysis and pharmacological potentials of different leaf extracts of *Aquilegia pubiflora*.

**Methods:**

Four types of extracts were prepared using solvent of different polarities (Distilled water AP_DW_, Methanol AP_M_, Ethanol AP_E_ and Ethyl acetate AP_EA_), and were evaluated to determine the best candidate for potent bioactivity. Phytochemical constituents in prepared extracts were quantified through HPLC analysis. Subsequently, all four types of leaf extracts were then evaluated for their potential bioactivities including antimicrobial, protein kinase inhibition, anti-inflammatory, anti-diabetic, antioxidant, anti-Alzheimer, anti-aging and cytotoxic effect.

**Results:**

HPLC analysis demonstrated the presence of dvitexin, isovitexin, orientin, isoorientin, ferulic acid, sinapic acid and chlorogenic acid in varied proportions in all plant extracts. Antimicrobial studies showed that, *K. pneumonia* was found to be most susceptible to inhibition zones of 11.2 ± 0.47, 13.9 ± 0.33, 12.7 ± 0.41, and 13.5 ± 0.62 measured at 5 mg/mL for AP_DW_, AP_M_, AP_E_ and AP_EA_ respectively. *A. niger* was the most susceptible strain in case of AP_DW_ with the highest zone of inhibition 14.3 ± 0.32, 13.2 ± 0.41 in case of AP_M_, 13.7 ± 0.39 for AP_E_ while 15.4 ± 0.43 zone of inhibition was recorded in case of AP_EA_ at 5 mg/mL. The highest antioxidant activity of 92.6 ± 1.8 μgAAE/mg, 89.2 ± 2.4 μgAAE/mg, 277.5 ± 2.9 μM, 289.9 ± 1.74 μM for TAC, TRP, ABTS and FRAP, respectively, was shown by AP_E_. AP_M_, AP_E_ and AP_EA_ extracts showed a significant % cell inhibition (above 40%) against HepG2 cells. The highest anti-inflammatory of the samples was shown by AP_E_ (52.5 ± 1.1) against sPLA2, (41.2 ± 0.8) against 15-LOX, followed by (38.5 ± 1.5) and (32.4 ± 0.8) against COX-1 and COX-2, respectively.

**Conclusions:**

Strong antimicrobial, Protein Kinase potency and considerable α-glucosidase, α-amylase, and cytotoxic potential were exhibited by plant samples. Significant anti-Alzheimer, anti-inflammatory, anti-aging, and kinase inhibitory potential of each plant sample thus aware us for further detailed research to determine novel drugs.

## Background

Phytochemicals are bioactive chemical compounds, which occur naturally in plants and are primarily responsible for protecting host plant by developing natural immune system, and provide specific aroma, flavor and color to the host plant. Traditionally used medicinal plants have gain ample attention in this era for development of novel drug compounds, as plants derived drugs and remedies have made a huge contribution to human safety, health and well-being [1]. The biologically active compounds derived from medicinal plants have demonstrated a great potential for treating human diseases such as cancer, heart disease, diabetes and infectious diseases [2]. Natural products have influenced several discoveries in organic chemistry, leading to advancement in synthetic methodologies by developing new lead compounds having pharmaceutical or therapeutic potential. Cinchona’s approval for treating malaria, preceded by digitalis and morphine and later the advent of aspirin, led the general population to believe in the miracles of natural floral resources [3].

The emergence of antibiotic resistance in some common pathogenic strains has substantially increased in the recent years, due to the consequence of indiscriminate use or misuse of antibiotics, hence results in escalating therapeutic problems [4]. Several medicinal plants and their bioactive products have been tested against antibiotic resistant pathogenic microbes [5]. The use of medicinal plants in food products in the form of bioactive compounds, such as carotenoids, flavonoids, phenolics and terpenoids etc. help to boost natural immunity of the body to fight against microbes [6–8]. These phytochemicals have been reported to perform different biological activities, e.g. anti-cancer [9], anti-inflammatory [10], antibacterial [11], anti-viral [12], anti-ischemic [13] and vasodilator [14].

Many medicinal plants possess large amounts of phytochemicals with antioxidant activities such as polyphenols, having ability to neutralize and quench free radicals, and are capable to decompose peroxides. These significant antioxidant properties possessed by phytochemicals are associated with prevention and treatment of several human disorders [15] Various researches have been conducted to isolate and characterize these compounds responsible for radical scavenging activities, to develop natural antioxidant formulations for cosmetics, medicine, and food industry [16–18].

In traditional medicines large number of medicinal herbs have been tested against inflammatory diseases and many of them are proved to be potent anti-inflammatory agents. A vast range of phytochemicals such as saponins, polysaccharides, lignans, anthraquinones, polyphenols, alkaloids, terpenoids, flavonoids are considered responsible for anti-inflammatory potential of the plants [19, 20]. However, their biochemical investigations has demonstrated that flavonoids are the major class of phytochemicals which acts as potent anti-inflammatory agents. The mode of action of different anti-inflammatory agents varies depending upon their chemical structures. Some of them acts as TNF-α inhibitors, while some inhibits phospholipases. It has been investigated that flavonoids blocks metabolism of arachidonic acid by inhibiting lipoxygenase and cyclooxygenase pathways [21, 22].

Several medicinal plants have anti-aging properties and have been used for delaying aging of body cells. Anti-aging potential of medicinal herbs is attributed to their ability to enhance vital energy levels in the body and to provide essential nutrients and modulate several pathological aspects because of their antimicrobial and anti-parasitic activities. Anti-aging medicinal herbs can promote health of all parts of the body including nerve cells and are effective against aging-associated neurological disorders [23–25].

The use of medicinal plants for treating cancer dates back to ancient times. Almost 3000 years ago Chinese practitioners prescribed herbal products as a remedy to cancer patients. The National Cancer Institute (USA) has successfully screened around 114,000 extracts of 35,000 medicinal plant samples collected from 20 countries for cancer treatment [26]. Various anti-cancer plant species have been reported, some of these plants includes *Fagonia indica* [27], *Linum usitatssimum* [28], *Cannabis sativa*, *Catharanthus roseus, Curcuma longa*, *Taxus baccata*, *Oroxylum indicum*, *Chelidonium majus,* and *Curcuma zedoaria* etc. [29]. Paclitaxel, a plant derived anti-cancer drug obtained from the bark extract of the Pacific Yew, is an evidence of use of natural plant products in drug discovery [30].

*Aquilegia publiflora* a medicinally important plant belongs to Genus Aquilegia and family ranunculaceae and is commonly known as Himalayan columbine. Plant species belonging to Ranunculaceae family contain pharmacologically important phytochemicals including *p*-coumaric acid, aquilegiolide apigenin, β sitosterol, ferulic acid, magnoflorine, resorcylic acid, genkwanin, glochidionolactone and caffeic acid [31–33]. Well known traditional medicinal applications of *Aquilegia pubiflora* include treatment of skin burns, hepatitis, wound healing, jaundice, circulatory and cardiovascular diseases [34–37].

To the best of our knowledge, it is the first ever report on HPLC analysis of medicinally important plant *Aquilegia pubiflora*. Different types of leaf extracts (Aqueous, methanol, ethanol and ethyl acetate) of *Aquilegia pubiflora* were prepared and were investigated for their biological applications.

## Methods

### Materials, reagents and strains

In the current research, all solvents used were of analytical grade and were supplied by Thermo Scientific (Illkirch, France). All reagents and standards were bought from Merck (Saint-Quentin-Fallavier, Lyon, France). Strains including *Bacillus subtilis* (ATCC 6633)*, Klebsiella pneumoniae* (ATCC 13883)*, Staphylococcus epidermidis* (ATCC 14490)*, Pseudomonas aeruginosa* (ATCC 9721), *Escherichia coli* (ATCC 15224), *Aspergillus flavus* (ATCC 9643)*, Aspergillus fumigatus* (FCBP 66), *Fusarium solani* (FCBP 434), *Aspergillus niger* (ATCC 1015) and *Mucor* species (FCBP 300) were acquired from Department of Biotechnology, QAU, Pakistan.

### Plant collection and extracts preparation

In this study the herb *Aquilegia pubiflora* was obtained from District Swat (Mian Damm), Khyber Pakhtunkhwa, Pakistan. The plant was taxonomically identified by the Department of Botany, Bacha Khan University, Charsadda as *Aquilegia Pubiflora* and was later confirmed by Professor Mushtaq Ahmad, Department of Plant Sciences, Quaid-i-Azam University Islamabad, Pakistan.

The fresh plant leaves were excised with a sterile surgical blade into tiny pieces, rinsed well under running tap water to eliminate any contaminants and impurities of soil, washed thrice with distilled water and were shade dried. The dried leaves were then ground into fine powder using a Willy mill and were processed for aqueous extraction at 25 °C. Aqueous, methanol, ethanol, and ethyl acetate extracts of plant leaves were prepared separately by adding 25 g of obtained powder in flasks (500 mL) comprising 200 mL of respective solvents, sonicated for 10 min, and were placed at 40 °C for 2 days in a shaking incubator at 200 rpm. The obtained extracts were initially filtered twice with nylon cloth to remove solid residues and were further filtered thrice using Whatman filter paper to remove any remaining particulates. The fresh filtrate was then dried and processed for further use.

### HPLC analysis

After aqueous extraction the obtained samples were analysed via HPLC using a separation technique adapted from [38]. HPLC standards were purchased from Sigma Aldrich. Here, an Hypersil PEP 300 C18, 250 × 4.6 mm, 5 μm particle size equipped with a guard column Alltech, 10 × 4.1 mm was used at 35 °C. Compound detection was achieved at 280 nm. The mobile phase was composed of a mixture of solvent A = HCOOH/H_2_O, pH = 2.1 and solvent B = CH_3_OH (HPLC grade solvents). The mobile phase composition varied during a 1 h run, with a nonlinear gradient as follows: 8% B (0 min), 12% B (11 min), 30% B (17 min), 33% B (28 min), 100% B (30–35 min) and 8% B (36 min) at a flow rate of 1 mL/min. A 10 min re-equilibration was applied between each run. Quantification was based on external 5-point Calibration curves with R^2^ of at least 0.998 using commercial reference standards (Sigma Aldrich). All the samples were analyzed three times and the results were expressed in μg/mg DW of the sample.

### Biological applications

#### Anti-bacterial assay

Agar disc diffusion method as reported previously [39], was employed to evaluate the antibacterial activity of test samples using concentrations ranging from 0.5 mg/L to 5 mg/L. Different bacterial strains used in this study include *Bacillus subtilis* (ATCC 6633)*, Klebsiella pneumoniae* (ATCC 13883)*, Staphylococcus epidermidis* (ATCC 14490)*, Pseudomonas aeruginosa* (ATCC 9721) and *Escherichia coli* (ATCC 15224). In brief, 50 μL of refreshed bacterial cultures were poured onto nutrient agar plates and were spread out uniformly with cotton swabs. Using a sterile borer, wells of 5 mm size were created, 10 μL of the tested samples were added in each well and the plates were labeled accordingly. In this assay, Ampicillin and DMSO were used as positive and negative control, respectively. The bacterial culture plates were then incubated for 24 h at 37 °C. After the incubation period, zones of inhibition obtained for each bacterial species were measured in mm using a Vernier caliper.

#### Anti-fungal assay

To test the fungicidal activity of prepared plant extracts, the samples were evaluated against five spore forming fungi including *Aspergillus flavus* (ATCC 9643)*, Aspergillus fumigatus* (FCBP 66), *Fusarium solani* (FCBP 434), *Aspergillus niger* (ATCC 1015) and *Mucor* species (FCBP 300), respectively. Briefly, the spore suspensions from stock cultures were prepared in Tween 20 solution (0.02% v/v) for each fungal strain. From each aliquot of stock suspension cultures, 100 μL volume was poured onto separate petri plates containing sterile SDA media and was swabbed well. Subsequently, tested samples (10 μL) were added into each well and the seeded plates were properly labelled. Ampicillin and DMSO were employed as positive and negative controls, respectively. The culture plates were then placed for 48 h incubation followed by examination and measurement of Zone of Inhibition to the nearest mm, using a Vernier caliper.

### Protein kinase inhibition assay

Protein kinase enzyme inhibition bioassay using different concentrations of prepared extracts ranging from 0.5 mg/L to 5 mg/L was performed following protocol [40] with slight modifications, to verify their protein kinase inhibitory ability, as a preliminary assay to screen the anti-cancerous potential of *Aquilegia pubiflora* leaf extracts. Briefly, Streptomyces 85E was employed as a test strain. A volume of 100 μL from the refreshed culture of Streptomyces 85E was added to the plates containing sterile ISP4 medium. Five millimetre size wells were created, filled with plant samples (5 μL) and were labeled accordingly. Surfactin and DMSO were employed as positive and negative controls, respectively. Subsequently, the culture plates were incubated at 28 °C for 48 h. The presence of clear and bald zones around the wells is an indication for inhibition of phosphorylation, mycelia and spore formation. A Vernier caliper was used to measure the zones to the nearest mm. Clear zones show the cytotoxic potential of respective extract sample via killing of the tested strain.

### In vitro α-amylase and α-glucosidase inhibition assays

Both ɑ-amylase and ɑ-glucosidase inhibition bioassays were performed to investigate the anti-diabetic potential of the samples.

### α-amylase inhibition assay

The protocol reported by [41], was followed with minor modifications to evaluate the α-amylase inhibition potential of test samples. Ninety-six well microplate was used for this assay. The phosphate buffer (15 μL), α-amylase (25 μL), test sample (10 μL) and starch (40 μL) were added to each well included in the test. The plate was then incubated for 30 min at 50 °C. Finally, 1 M HCl (20 μL) and 90 μL of iodine solution were added into each well. DMSO and acarbose served as negative and positive controls while blank contained buffer solution and starch instead of *Aquilegia pubiflora* leaf extracts. Absorbance was recorded at 540 nm using a microplate reader. The inhibition was calculated as percentage using the formula

$$ \%\mathrm{Enzyme}\ \mathrm{inhibition}=\left(\frac{\mathrm{Abs}\ \mathrm{Sample}-\mathrm{Abs}\ \mathrm{negative}\ \mathrm{control}}{\mathrm{Abs}\ \mathrm{blank}-\mathrm{Abs}\ \mathrm{negative}\ \mathrm{control}}\right)\times 100 $$

### α-glucosidase inhibition assay

The anti-diabetic potential of extracts was further determined by α-glucosidase inhibition bioassay using a previously reported protocol with minute modifications [42, 43]. In the experiment, 50 mL of phosphate buffer (pH 6.8) supplemented with 100-mg BSA (bovine serum albumin) was used to dissolve α-glucosidase (*Saccharomyces cerevisiae*, Sigma-Aldrich). Reaction mixtures constituting 10 μL of tested sample, phosphate buffer (490 *μ*L; pH 6.8) and *p*-nitrophenyl α-D-glucopyranoside (5 mM; 250 μL) were kept sepatately for incubation at 37 °C for 5 min. Two hundred fifty microlitre α-glucosidase (0.15 unit/mL) was then added to each mixture followed by incubation for 15 min at 37 °C. After terminating reaction, by adding 2 mL Na_2_CO_3_ (200 mM) solution, absorption was recorded using a UV-Vis spectrophotometer at 400 nm. The assay is based on the quantification of *p*-nitrophenol release from *p*-nitrophenyl α-D-glucopyranoside. In the experiment, acarbose was employed as a positive control and assay was repeated three times.

$$ \%\mathrm{Enzyme}\ \mathrm{inhibition}=\left(\frac{Abs\  Sample- Abs\  negative\ control}{Abs\  blank- Abs\  negative\ control}\right)\times 100 $$

### Antioxidant assays

#### Total Antioxidant Capacity determination (TAC)

The assay reported by [39], was employed to examine the total antioxidant capability of the samples. In the experiment, 100 μL of sample was added to the Eppendorf tubes with the help of micropipette. After that, 900 mL of TAC reagent (0.6-M sulfuric acid, 28 mM sodium phosphate and 4 mM ammonium molybdate, in 50 mL dH_2_0) was transferred to Eppendorf tubes containing the tested samples. The reaction mixture was placed in a water bath for incubation at 90 °C for 2.5 h followed by cooling at room temperature. The absorbance of the samples was then measured at 630 nm through a microplate reader. The experiment was performed three times and TAC was expressed as μg ascorbic acid equivalent per milligram of the sample.

#### Total Reducing Power determination (TRP)

The procedure reported by [39], was used in triplicate to check the total reducing power of the sample. Each test sample (100 μL) along with 400 μL of 0.2 Molar phosphate buffer (pH 6.6) and potassium ferric cyanide (1% w/v) was added to the Eppendorf tubes followed by incubation in a water bath at 55 °C for 30 min. Subsequently, 400 μL of trichloroacetic acid (10% w/v) was added to each Eppendorf tube followed by centrifugation for 10 min at 3000 rpm. The supernatant (140 μL) of each mixture was poured into corresponding wells of a 96-well plate containing 60 μL of ferric cyanide solution (0.1% w/v). The absorbance of the samples was then recorded using a microplate reader at 630 nm. The same procedure, as mentioned earlier was followed both for positive and negative controls. Total reducing power (TRP) of was expressed as (μg ascorbic acid equivalent) per milligram the tested sample.

#### Free Radical Scavenging Assay (FRSA)

The protocol reported previously by [44, 45], was adopted with minor changes. The possible free radical scavenging ability of test samples was investigated for their antioxidant potential using DPPH reagent at concentrations ranging from 12.5 μL to 400 μL. Tested samples (10 μL) were added to each well of a 96-well plate. DPPH reagent (190 μL) was then transferred to each well having the sample. The plates were incubated in the dark for 60 min at 37 °C. Ascorbic acid was used as a positive control and DMSO was employed as a negative control. Absorbance of reaction mixture was measured at 515 nm using a microplate reader and free radical scavenging potential was measured in percentage using the following equation

$$ \left(\%\right)\ \mathrm{FRSA}=\left(1-\frac{Abs}{Abc}\ \right)\times 100 $$

Where Ab_c_ and Ab_s_ indicate the absorbance of the negative control and sample, respectively.

#### ABTS assay

ABTS scavenging assay also known as (Trolox antioxidant assay) was tested using [46] protocol with slight modifications. ABTS reaction solution was prepared by combining potassium per sulfate (2.45 mM) with 7 mM ABTS salt in the same proportion followed by 16 h dark incubation. At 25 °C, the final reaction mixture was prepared by adding test samples in the above prepared mixture and allowed the reaction to proceed by keeping in dark for 15 min. Test samples absorbance was recorded via Microplate Reader (BioTek ELX800) at 734 nm. Trolox and DMSO were used as positive and negative control. The samples’ antioxidant potential was represented as TEAC, and assays were performed in triplicate manner.

#### In vitro AChE and BChE inhibition assays

Antialzheimers activity of plant extracts were measured by their ability to inhibit acetylcholinesterase (AChE; Sigma “101,292,679: 0.03 U/mL) and butyrylcholinesterase (BChE; Sigma “101,303,874: 0.01 U/mL). In brief, leaf extracts were dispersed in phosphate buffer saline (PBS) with a concentration ranging from 25 μg/mL to 400 μg/mL. A substrate solution was prepared in distilled water that constituted butyrylcholine iodide (BTchI; 0.0005 M), DTNB (5, 5-dithiobisnitrobenzoic acid; 0.00022 M), and acetylcholine iodide (ATchI; 0.0005 M). In the assay, pristine reaction mixture and Galanthamine hydrobromide (5 mg/0.5 mL methanol) were used as positive and negative controls, respectively. The principle of the assay is based on ATchI and BTchI hydrolysis by AChE and BChE, respectively, leading to 5- thio-2- nitrobenzoate anion formation that gives a yellow colour when form complexes with DTNB [47]. Finally, the absorbance of the samples was recorded using a UV-VIS spectrophotometer at 412 nm. The percentage enzyme activity and enzyme inhibition activities were calculated as

$$ \%\mathrm{Enzyme}\ \mathrm{activity}=\left(\frac{V}{V\ \mathit{\max}}\right)\times 100 $$

$$ \%\mathrm{Enzyme}\ \mathrm{inhibition}=100-\% Enzyme\ activity $$

### Anti-inflammatory activities

#### Inhibitory activity against COX-1 and COX-2

The inhibitory potential of test samples was tested against COX-1 (Ovine kit 701,050 France) and COX-2 (Human kit 701,050 France). Ibuprofen 10 μM was used as a positive control and arachidonic acid was used as a substrate at 1.1 mM concentration. Both the COXs were measured by following the manufacturer’s instructions on kit. The assay was conducted in triplicate in a 96-well plate. Synergy II reader was used at 590 nm to check N, N, N^/^, N^/^−tetramethyl-p-phenylenediamine in 96-well microplate.

#### Inhibitory activity against 15-LOX

The inhibitory potential of plant extracts against 15-LOX (760,700 kit, Cayman France) was performed. Nordihydroguaiaretic acid (NDGA) 100 μM was used as positive control while 10 μM arachidonic acid was taken as a substrate. Lipooxygenation reaction occurs, which produces hydroperoxides whose concentration was measured by soy 15-lipooxygenase standard in 10 mM Tris-HCl buffer at 7.4 pH filter fitted in the kit. The test samples and enzyme are poured in 96-well plate, incubated for 5 min and the absorbance was measured at 940 nm using Synergy II reader (BioTek Instruments, Colmar, France). The inhibitor was added in the enzyme mixture, incubated for 5 min and absorbance was recorded. Then substrate was added in the preincubated mixture and in last chromogen was added in the mixture and absorbance was measured after 5 min of incubation.

#### Inhibitory activity against secretory phospholipase A2 (sPLA2)

Assay kit (10,004,883, Cayman Chem. Co, Interchim, Montluçon, France) was used to check the inhibitory potential of samples against sPLA2. One point foryt-four millimetre diheptanoyl thio-PC and 100 μM thiotheramide-PC served as positive control and substrate, respectively. The cleavage of diheptanoyl thio-PC ester releases free thiols which were measured by Synergy II reader (BioTek Instruments, Colmar, France) at 420 nm in a 96-well microplate using DTNB (5–50-dithio-bis-(2-nitrobenzoic acid). The percentage inhibition was calculated as.

$$ \%\mathrm{inhibition}=\left[\left(\mathrm{IA}-\mathrm{inhibitor}\right)/\mathrm{IA}\right]\ast 100 $$

### Anti-aging assay

#### Anti-AGE formation activity

The inhibitory potential of leaf extracts against Vesperlysine AGEs and Pentosidine AGE formation was measured by the previously described protocol of [48]. For this purpose, BSA (Sigma Aldrich) solution was prepared using 0.5 M glucose (Sigma Aldrich) solution and 0.1 M phosphate buffer (pH 7.4), containing 0.02% (w/v) sodium azide. *Aquilegia pubiflora* leaf extracts were mixed with 20 mg/mL BSA solution. The reaction mixture was dark incubated for 5 days at 37 °C. VersaFluor fluorometer; Bio-Rad, Marnes-la-Coquette, France set was used to record the amount of fluorescent producedby taking absorbance at 330 nm of excitation wavelength and 410 nm emission wavelength, respectively.

#### Tyrosinase assay

The previously described method of [49], using L-DOPA (5 mM; Sigma Aldrich) was used for performing tyrosinase assay. L-DOPA diphenolase substrate was mixed with 10 μL of test sample along with sodium phosphate buffer (50 mM, pH 6.8). The final volume of the reaction mixture was raised to 200 μL by adding 0.2 mg/mL of mushroom tyrosinase solution (Sigma Aldrich). The extraction solvent replacing the tested sample was used as control. Microplate reader (BioTek ELX800; BioTek Instruments) was used to trace the reaction processes at 475 nm. Relative to corresponding control tyrosinase effect was expressed as percent inhibition.

#### Elastase assay

Porcine pancreatic elastase (Sigma Aldrich) was used for the determination of elastase inhibition. N-Succ-Ala-Ala-Ala-p-nitroanilide (AAAVPN; Sigma Aldrich) served as a substrate in this experiment. The reaction OD was calculated from the relative conversion of the substrate into *p*-nitroaniline at 410 nm using a microplate reader (BioTek ELX800; BioTek Instruments) [50]. The assay was conducted three times and the anti-elastase potential was expressed as a percentage inhibition relative to the corresponding power.

#### Hyaluronidase assay

The protocol developed by [51], was used for assessing the hyaluronidase inhibitory potential of test samples. A solution containing hyaluronic acid (0.03% (w/v)) and 1.5 units of hyaluronidase (Sigma Aldrich) was used as a substrate. The precipitation of the undigested form of hyaluronic acid occurred with acid albumin solution (0.1% (w/v) BSA). OD was calculated at 600 nm using a microplate reader (BioTek ELX800; BioTek Instruments, Colmar, France). Relative to the corresponding control the anti-hyaluronidase potential was expressed as a percent inhibition.

#### Collagenase assay

Collagenase inhibitory assay was performed following a reported the protocol of [50] with slight modification. [(2-furyl) acryloyl]-Leu-Gly-Pro-Ala (FALGPA; Sigma Aldrich) worked as a substrate in this assay. The decline in FALGPA absorbance was continuously observed for 20 min by recording OD at 335 nm using a microplate reader (BioTek ELX800; BioTek Instruments, Colmar, France). The assay was performed in triplicate manner and relative to control anti-collagenase was shown as percent inhibition.

#### Anti-leishmanial assay

The anti-leishmanial potential of the plant extracts was assessed against the amastigote and promastigote cultures of *L. tropica* KWH23 (Department of Biotechnology IIUI Pakistan) [46, 52]. M199 media having 10% fetal bovine serum was used for culturing of leishmanial parasites. *Leishmania* culture at a density of 1 × 10^6^ cells/ml was used for the analysis. The activity was performed in a 96-well plate with a concentration ranging from 400 to 25 μg/mL. DMSO was used as a blank and Amphotericin served as a positive control in the experiment. The seeded 96-well plate with test dilutions was incubated at room temperature for 72 h. OD was noted at 540 nm, while all lived cultures were counted using an inverted microscope and their LC50 values were calculated by using Table curve software. Percent inhibition was measured as

$$ \%\mathrm{Inhibition}=\left[1-\left\{\frac{\mathrm{Absorbance}\ \mathrm{of}\ \mathrm{sample}}{\mathrm{Absorbance}\ \mathrm{of}\ \mathrm{control}}\right\}\right]\times 100 $$

#### Cytotoxicity against HepG2 cell line

HepG2 cells (ATCC HB-8065) were grown in Dulbecco’s Modified Eagle Medium (DMEM) supplemented with 10% of Fetal calf serum (FCS), 100 U/mL penicillin, 2 mM L-glutamine, 100 μg/mL streptomycin and 1 mM Na-pyruvate and were incubated in a 5% humified CO_2_ atmosphere at 37 °C. The confluent cell layer was harvested using 0.5 mM trypsin/EDTA. MTT (3–4, 5-dimethylthiazol-2-yl) − 2, 5-diphenyltetrazolium bromide), a tetrazolium dye was used to access the cytotoxic potential of different leaf extracts in vitro. In this assay,, MTT become reduced into its insoluble purple product formazan which is measured spectrophotometrically. In a 96-well plate, pre-seeded HepG2 cells (> 90% viability; 1 × 10^4^ cells/well or 10,000 cells per well) were treated with 200 μg/mL of test samples for 24 h. Later, 10 μL of MTT dye (5 mg/mL) was added per well, followed by incubation of 3 h. Insoluble formazan was then dissolved by adding 10% acidified sodium dodecyl sulfate (SDS). Cells were then incubated overnight. Plates were analyzed at 570 nm using a microplate reader (Platos R 496, AMP). Non-treated HepG2 cells (NTC) were included as control. DMSO was used as a negative control for plant extracts. Optical Density of treated samples and NTC was measured at 570 nm. Percent (%) viability was calculated relative to the NTC sample using the following formula:

$$ \%\mathrm{Viability}=\frac{\mathrm{Absorbance}\ \mathrm{of}\ \mathrm{sample}-\mathrm{Absorbance}\ \mathrm{of}\ \mathrm{sample}\ \mathrm{control}}{\mathrm{Absorbance}\ \mathrm{of}\ \mathrm{NTC}-\mathrm{Absorbance}\ \mathrm{of}\ \mathrm{media}} \times 100 $$

## Results

### HPLC analysis of different plant extracts

**Fig. 1:**
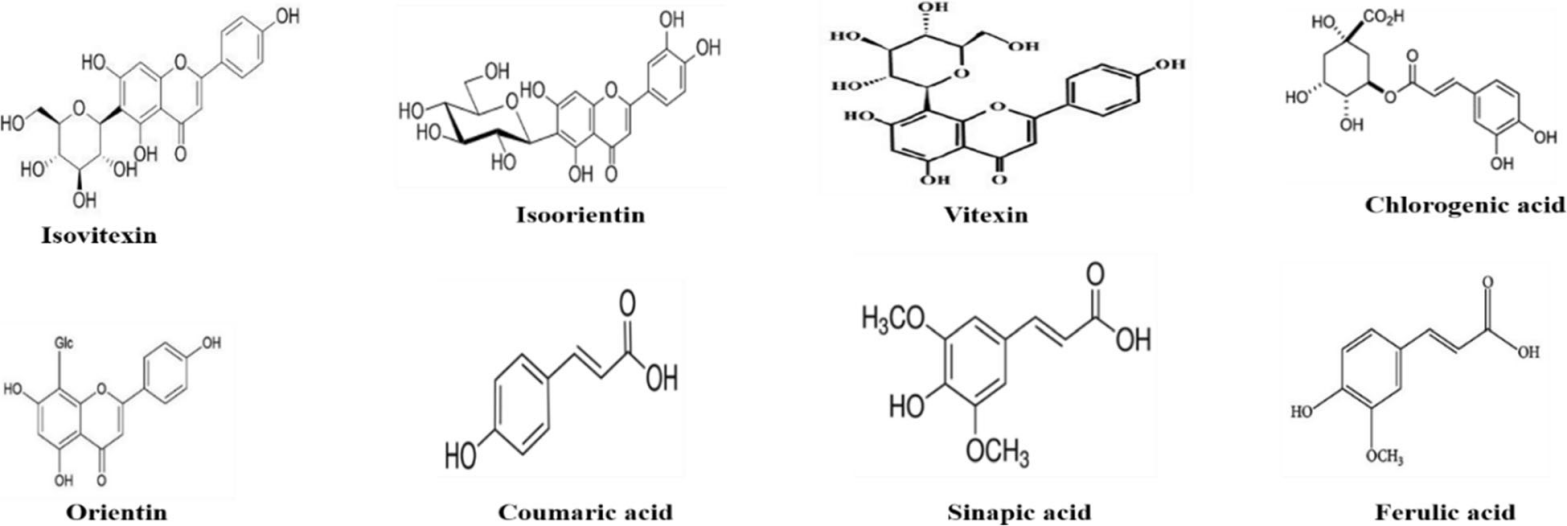
Bioactive compounds detected in *Aquilegia pubiflora* leaf extracts

Genus Aquilegia belongs to the *Ranunculaceae* family, which has more than 60 species of plants that are used primarily in South Asia and worldwide for many medicinal purposes. These plants have abundant phytochemicals with efficient therapeutic properties [31–33]. In this study, *Aquilegia publiflora* leaf extracts were prepared using four solvents with different polarities. To determine and quantify the specific phytochemicals present in these extracts, high-performance liquid chromatography (HPLC) was performed. Eight compounds, including four flavonoids (vitexin, orientin, isovitexin, and isoorientin) and four derivatives of hydroxycinnamic acid (chlorogenic acid, ferulic, sinapic acid and *p*-coumaric acid) were identified (based on their retention times and UV spectra compared to commercial standards) and quantified for all leaf extracts (Fig. 1). Flavonoids and hydroxycinnamic acids are phenylpropanoids generated via the pathway of shikimic acids and have been correlated with various biological activities of medicinal plants [53]. HPLC results indicated a higher number of flavonoids and derivatives of hydroxycinnamic acid as shown in Table 1. Among the detected flavonoids orientin was observed to be present in a higher concentration for AP_EA_ and AP_E_ (171 ± 2.4 μg/g DW and 983 μg/g DW respectively) while chlorogenic acid was found to be abundant hydroxycinnamic acid for both AP_EA_ and AP_E_ (1.15 ± 0.08 μg/g DW and 1.70 μg/g DW respectively). Orientin and chlorogenic acid protect the plant from stress conditions and perform various biological activities such as, antioxidant, anti-aging, anti-inflammatory, anti-diabetic, antifungal, antibacterial, hepatoprotective and anticancer [54].

**Table 1:** HPLC analysis of *Aquilegia pubiflora* leaf extract in different solvents

Phytochemicals	AP_DW_ (μg/g DW)	AP_M_ (μg/g DW)	AP_E_ (μg/g DW)	AP_EA_ (μg/g DW)
FLAVONOIDS				
Orientin	72.56 ± 3.09	63.30 ± 2.7	983 ± 4.3	171 ± 2.4
Isoorientin	54.03 ± 2.02	37.05 ± 1.6	483.79 ± 4.1	97.33 ± 1.90
Isovitexin	16.982 ± 2.65	7.719 ± 0.63	161.78 ± 2.9	48.27 ± 1.1
Vitexin	92.63 ± 3.71	58.66 ± 1.91	489.96 ± 3.8	86.23 ± 1.21
HYROXYCINNAMIC ACIDS				
*p*-Coumaric acid	0.069 ± 0.011	0.049 ± 0.007	0.31 ± 0.3	0.26 ± 0.2
Ferulic acid	0.145 ± 0.014	0.076 ± 0.006	0.52 ± 0.7	0.44 ± 0.5
Sinapic acid	0.049 ± 0.007	0.038 ± 0.007	0.20 ± 0.4	0.16 ± 0.3
Chlorogenic acid	0.355 ± 0.13	0.280 ± 0.006	1.70 ± 0.11	1.15 ± 0.08

### Antibacterial activity

Antibiotic resistance is a serious issue that tends to plague the healthcare system of both emerging and industrialized countries around the globe [55]. The advent and dissemination of multidrug-resistant infections has affected the existing antibacterial treatments greatly. Therefore, a quest for new sources of antimicrobial agents has increased in recent years, to combat with the resistant infectious pathogens. In this context, medicinal plants with antimicrobial potential have been widely explored, as they contain a range of bioactive compounds with proven therapeutic properties [56–58]. This study was aimed to evaluate the antimicrobial activity of medicinal plant *Aquilegia pubiflora* extracts against five pathogenic bacteria, including 2 Gram-positive (*Staphylococcus epidermidis* and *Bacillus subtilis*) and 3 Gram-negative (*Escherichia coli, Klebsiella pneumoniae,* and *Pseudomonas aeruginosa*) using well disc diffusion method. These organisms were frequently encountered in infectious diseases [59]. The research demonstrated that all plant extracts used had varied degrees of antimicrobial activity against all microorganisms tested at five different concentrations (5 mg/mL, 4 mg/mL, 2 mg/mL, 1 mg/mL, and 0.5 mg/mL). All the tested strains were susceptible to all extracts shown in (Table 2) with *K. pneumonia* is found to be most susceptible. Inhibition zones measured at 5 mg/mL for AP_DW_, AP_M_, AP_E_ and AP_EA_ were 11.2 ± 0.47, 13.9 ± 0.33, 12.7 ± 0.41, and 13.5 ± 0.62 for *Klebsiella pneumoniae,* respectively. Our findings was close to that of previous studies that documented antibacterial activity of methanolic extract of *O. corniculata* [60]. AP_DW_ and AP_E_ inhibited *S. epidermidis* and *E. coli* at concentration-dependent manner however, highest zone of inhibition was observed against *S. epidermidis* (12.3 ± 0.21) and *E. coli* (12.6 ± 0.29) for AP_E_ at 5 mg/mL, respectively. In another study, aqueous and ethanolic extract from *C. tamala* plant was found to have the same antimicrobial activity against the tested bacterium, *S. aureus* and *K. pneumoniae* [61]. Furthermore, *E. coli* was the most susceptible strain to AP_EA_ with significant zone of inhibition (11.7 ± 0.31) followed by *S. epidermidis* (11.3 ± 0.39) at 5 mg/mL concentration.

**Table 2:** Zones of inhibitions of plant extracts against bacterial strains at different concentrations

Bacterial Strains	Sample	Concentration					
		Ampicillin	5 mg/mL	4 mg/mL	2 mg/mL	1 mg/mL	0.5 mg/mL
		Zone of inhibition in (mm)					
*Bacillus subtilis*	AP_DW_	15.4 ± 0.67	10.1 ± 0.20*	8.2 ± 0.31**	8.1 ± 0.27**	6.4 ± 0.19***	5.7 ± 0.19***
	AP_M_	17.1 ± 0.74	12.1 ± 0.38*	11.7 ± 0.34*	10.2 ± 0.29**	7.3 ± 0.24***	6.2 ± 0.17***
	AP_E_	15.8 ± 0.72	12.2 ± 0.39*	9.3 ± 0.32*	6.9 ± 0.22**	6.1 ± 0.18**	4.7 ± 0.21***
	AP_EA_	15.5 ± 0.69	10.7 ± 0.43*	9.7 ± 0.26*	6.3 ± 0.21**	5.7 ± 0.19***	5.2 ± 0.31***
*Staphylococcus epidermidis*	AP_DW_	16.4 ± 0.59	10.3 ± 0.27*	10.1 ± 0.39*	7.3 ± 0.19**	6.2 ± 0.25**	4.4 ± 0.17***
	AP_M_	17.3 ± 0.57	10.4 ± 0.22*	8.6 ± 0.29*	6.9 ± 0.24**	5.2 ± 0.17***	4.1 ± 0.18***
	AP_E_	15.4 ± 0.40	12.3 ± 0.21*	10.2 ± 0.44**	10.2 ± 0.37**	8.3 ± 0.31**	5.4 ± 0.24***
	AP_EA_	14.8 ± 0.38	11.3 ± 0.39*	8.4 ± 0.31*	6.6 ± 0.28**	6.2 ± 0.19**	4.8 ± 0.17***
*Klebsiella pneumoniae*	AP_DW_	15.9 ± 0.33	11.2 ± 0.47*	10.7 ± 0.42*	8.3 ± 0.37**	7.1 ± 0.29**	5.4 ± 0.19***
	AP_M_	16.9 ± 0.61	13.9 ± 0.33*	11.4 ± 0.45*	9.1 ± 0.33**	6.9 ± 0.32***	6.2 ± 0.23***
	AP_E_	15.6 ± 0.39	12.7 ± 0.41*	10.6 ± 0.39*	7.7 ± 0.32**	5.8 ± 0.27***	5.2 ± 0.22***
	AP_EA_	15.2 ± 0.34	13.5 ± 0.62*	12.2 ± 0.53*	9.4 ± 0.32*	8.1 ± 0.29**	6.7 ± 0.28**
*Escherichia coli*	AP_DW_	15.7 ± 0.41	10.8 ± 0.31*	8.3 ± 0.31*	7.0 ± 0.19**	6.3 ± 0.16**	4.8 ± 0.19***
	AP_M_	17.2 ± 0.66	12.9 ± 0.59*	12.1 ± 0.51*	9.5 ± 0.35*	7.1 ± 0.28**	5.4 ± 0.19***
	AP_E_	15.9 ± 0.54	12.6 ± 0.29*	11.2 ± 0.49*	8.4 ± 0.28**	6.7 ± 0.24***	6.0 ± 0.21***
	AP_EA_	15.5 ± 0.48	11.7 ± 0.31*	10.3 ± 0.34*	9.2 ± 0.19*	7.2 ± 0.21**	5.1 ± 0.22***
*Pseudomonas aeruginosa*	AP_DW_	16.2 ± 0.46	11.1 ± 0.29*	9.2 ± 0.47*	7.7 ± 0.43**	5.9 ± 0.28***	4.3 ± 0.21***
	AP_M_	16.3 ± 0.57	13.6 ± 0.61*	10.1 ± 0.33*	10.4 ± 0.28*	8.4 ± 0.29**	6.9 ± 0.21**
	AP_E_	15.4 ± 0.44	12.1 ± 0.29*	11.3 ± 0.41*	8.4 ± 0.44*	6.1 ± 0.32**	4.8 ± 0.19***
	AP_EA_	15.9 ± 0.41	11.1 ± 0.22*	9.9 ± 0.33**	7.4 ± 0.39**	7.1 ± 0.27**	5.4 ± 0.19***

Star *–*** represent; *** highly significant, ** slightly significant and * non-significant difference from control at *P* < 0.05 by one-way ANOVA in the column. Values are mean ± SD of triplicate. Where AP_DW_, AP_M_, AP_E_ and AP_EA_ stands for Distilled water, Methanol, Ethanol and Ethyl acetate extracts, respectively

### Antifungal assay

The anti-fungal activity of plants may be attributed to the presence of antifungal toxicants in their extracts. The fungicidal behavior has also been documented by several authors in a large variety of taxa. The existence of antifungal compounds in higher plants is well known and considered useful for preventing plant diseases [62]. Five pathogenic fungal strains (*A. fumigatus, A. flavus, M. racemosus, F. solani and A. niger*) were tested against *Aquilegia pubiflora* leaf extracts using the well diffusion method at concentrations in the range of 0.5 mg/mL to 5 mg/mL. The results presented in Table 3, clearly demonstrated that *A. niger* was the most susceptible strain in case of AP_DW_ with the highest zone of inhibition 14.3 ± 0.32, 13.2 ± 0.41 in case of AP_M_, 13.7 ± 0.39 for AP_E_ while 15.4 ± 0.43 zone of inhibition was recorded in case of AP_EA_ at 5 mg/mL, respectively. Previously, plant extracts from *Ageratum conyzoides* were found to have the same antifungal activity against the tested fungal specie [63]. Furthermore, these extracts *(*AP_E_ and AP_EA_) showed impressive fungicidal activity of (13.2 ± 0.52 and 14.8 ± 0.40) as shown in (Table 3), against *F. solani* at 5 mg/mL concentration. These obtained results are in accordance with the previously reported studies that revealed efficient antifungal activity of plant extracts [64]. Among the tested strains, *A. flavus* and *M. racemosus* were less inhibited strains in case of AP_M_ and AP_E_ at all concentrations. Plant extract AP_DW_ seemed to be effective against *M. racemosus, F. Solani* and *A. flavus* with zone of inhibition 14.1 ± 0.32, 13.8 ± 0.71 and 13.2 ± 0.52, respectively. In a conclusion, all types of leaf extracts have exhibited efficient fungicidal activity against fungal strains *A. niger, M. racemosus* and *F. solani* at concentration-dependent manner.

**Table 3:** Zones of inhibitions of plant extracts against fungal strains at different concentrations

Fungal Strains	Sample	Concentration					
		Ampicillin	5 mg/mL	4 mg/mL	2 mg/mL	1 mg/mL	0.5 mg/mL
		Zone of inhibition in (mm)					
*Aspergillus niger*	AP_DW_	18.1 ± 0.73	14.3 ± 0.32*	10.2 ± 0.42*	7.4 ± 0.25**	5.3 ± 0.21***	4.1 ± 0.19***
	AP_M_	17.6 ± 0.74	13.2 ± 0.41*	11.3 ± 0.31*	8.1 ± 0.28*	6.4 ± 0.19**	5.2 ± 0.31***
	AP_E_	17.7 ± 0.61	13.7 ± 0.39*	9.6 ± 0.51*	6.8 ± 0.37**	4.8 ± 0.27***	4.0 ± 0.31***
	AP_EA_	19.4 ± 0.43	15.4 ± 0.43*	11.3 ± 0.52*	7.7 ± 0.31**	6.9 ± 0.22**	5.7 ± 0.21***
*Aspergillus fumigatus*	AP_DW_	17.4 ± 0.59	13.5 ± 0.45*	12.2 ± 0.44*	9.3 ± 0.33*	7.1 ± 0.28**	6.3 ± 0.19**
	AP_M_	17.9 ± 0.57	12.7 ± 0.32*	9.4 ± 0.23**	6.4 ± 0.26**	5.1 ± 0.21***	4.7 ± 0.21***
	AP_E_	17.4 ± 0.40	13.0 ± 0.66*	10.7 ± 0.57*	8.5 ± 0.34**	6.4 ± 0.24***	6.0 ± 0.21***
	AP_EA_	19.8 ± 0.38	14.2 ± 0.61*	11.9 ± 0.51*	8.7 ± 0.39**	6.3 ± 0.21**	4.9 ± 0.21***
*Fusarium solani*	AP_DW_	17.9 ± 0.33	13.8 ± 0.71*	12.1 ± 0.34*	7.4 ± 0.22**	6.1 ± 0.17**	5.3 ± 0.28***
	AP_M_	18.1 ± 0.61	12.9 ± 0.61*	9.6 ± 0.32*	6.3 ± 0.21**	4.8 ± 0.27***	4.3 ± 0.19***
	AP_E_	16.2 ± 0.52	13.2 ± 0.52*	11.4 ± 0.51*	7.7 ± 0.29**	6.3 ± 0.22**	3.8 ± 0.16***
	AP_EA_	18.8 ± 0.40	14.8 ± 0.40*	12.1 ± 0.47*	9.1 ± 0.41*	6.6 ± 0.28***	5.1 ± 0.21***
*Mucor racemosus*	AP_DW_	18.7 ± 0.41	14.1 ± 0.32*	12.3 ± 0.29*	6.9 ± 0.38**	4.6 ± 0.21***	3.7 ± 0.18***
	AP_M_	17.7 ± 0.66	12.6 ± 0.49*	11.1 ± 0.39*	8.3 ± 0.31**	6.8 ± 0.28***	5.2 ± 0.19***
	AP_E_	17.9 ± 0.54	13.2 ± 0.54*	9.9 ± 0.49*	7.2 ± 0.32*	5.8 ± 0.27**	4.4 ± 0.21***
	AP_EA_	18.5 ± 0.48	13.9 ± 0.39*	11.2 ± 0.51*	9.1 ± 0.30*	6.6 ± 0.27**	4.1 ± 0.19***
*Aspergillus flavus*	AP_DW_	18.2 ± 0.64	13.2 ± 0.52*	10.8 ± 0.49*	8.2 ± 0.33**	6.9 ± 0.31***	5.1 ± 0.28***
	AP_M_	18.4 ± 0.72	12.0 ± 0.59*	8.9 ± 0.37**	6.7 ± 0.22**	6.2 ± 0.18**	3.8 ± 0.16***
	AP_E_	18.1 ± 0.40	12.4 ± 0.51*	9.1 ± 0.34*	7.4 ± 0.29**	5.4 ± 0.23***	5.4 ± 0.17***
	AP_EA_	19.2 ± 0.74	13.0 ± 0.37*	10.3 ± 0.29*	7.1 ± 0.22**	5.6 ± 0.26***	4.1 ± 0.18***

Star *–*** represent; *** highly significant, ** slightly significant and * non-significant difference from control at *P* < 0.05 by one-way ANOVA in the column. Values are mean ± SD of triplicate. Where AP_DW_, AP_M_, AP_E_ and AP_EA_ stands for Distilled water, Methanol, Ethanol and Ethyl acetate extracts, respectively

### Protein kinase inhibition assay

**Fig. 2:**
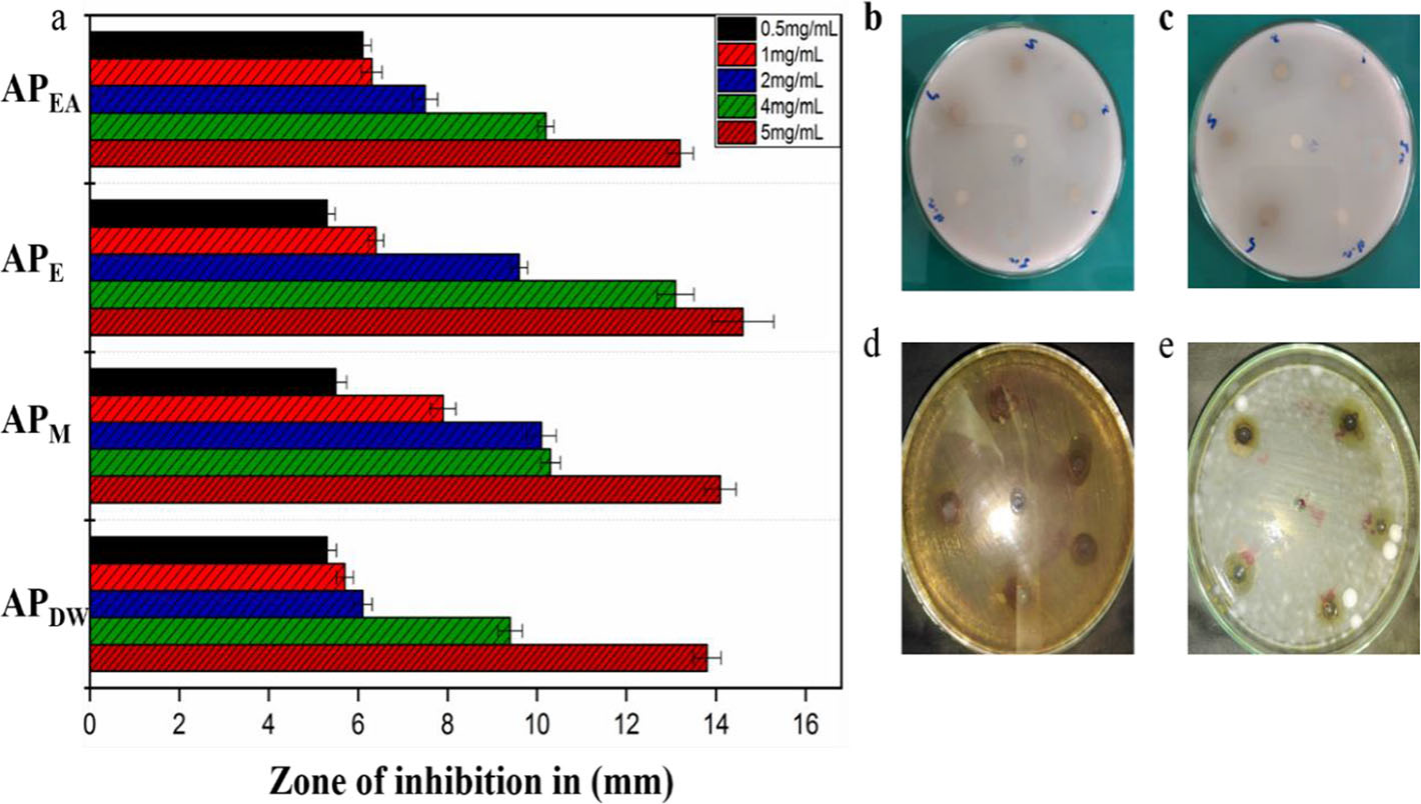
**a** Protein kinase inhibitory potential, **b** image for AP_E_ kinase inhibition against Streptomyces 85E, **c** image for AP_M_ kinase inhibition against Streptomyces 85E, **d** image for AP_E_ use against *K. pneumonia*, **e** image for AP_E_ use against *A. niger*. Values are mean ± SD of triplicate. Where AP_DW_, AP_M_, AP_E_ and AP_EA_ stands for Distilled water, Methanol, Ethanol and Ethyl acetate extracts, respectively

Protein kinase inhibitors are a well-established class of clinically useful drugs, particularly for treating cancer [65]. For particular protein kinases, achieving inhibitor selectivity remains a significant challenge, in order to use them as a tool for chemical biology research or in the development of new small molecules as drugs [66, 67]. These enzymes phosphorylate serine-threonine and tyrosine amino acid residues that have a key function in cellular proliferation, differentiation and apoptosis [68]. Deregulated phosphorylation by protein kinase can lead to tumor growth and entities with capability to inhibit these enzymes, are important area in anticancer research [69]. Streptomyces 85E strain was used to check the protein kinase inhibition potential of *Aquilegia pubiflora* extracts. Results as indicated in Fig. 2a showed that no clear zones were observed against each tested concentration of prepared extracts. Comparatively, AP_E_ showed the largest bald zone 14.6 ± 0.69 than AP_M_ 14.1 ± 0.35, AP_DW_ 13.8 ± 0.31 and AP_EA_ 13.2 ± 0.29 at 5 mg/mL. Lowest 5.3 ± 0.21 zone appeared in case of AP_DW_ 0.5 mg/mL representing least potential against Streptomyces hyphae growth. All the tested plant extracts inhibited Streptomyces strain at concentration-dependent manner as indicated in Fig. 2a. Overall, results showed that the entire test samples acquire vital metabolites responsible for anti-cancerous potentials in *Aquilegia pubiflora*. Our results are strongly supported by a previous report, which was conducted on the evaluation of hyphae formation inhibition in *Streptomyces* 85E. The isolated compounds in this reported study had showen impressive zone of inhibition at 80 μg/disk and it was hypothesized that the compounds prevent the formation of hyphae in *Streptomyces* 85E, which may inhibit cancer proliferation [70].

### In vitro α-amylase and α-glucosidase inhibition assays

Diabetes is a lifelong systemic disease induced by insulin secretion deficiency. Type 2 diabetes is more common of the two groups [71]. Some therapies used for treatment of type 2 diabetes includes the regulation of blood sugar and it can be achieved by preventing the absorption of glucose by inhibiting the enzymatic action of α-glucosidase and pancreatic α-amylase [72, 73]. Natural products, particularly plant products, are the key quarry for the discovery of promising lead candidates and play an imperative role in the upcoming drug development programs, mainly in the field of diabetes and cancer [74]. Ease of delivery, low expense and least side effects render plant-based preparations as themost accessible therapies, especially in rural areas [75]. In fact, many plants have a rich supply of bioactive chemicals, free of undesirable side effects and have effective pharmacological potential against diabetes [76, 77]. Plants have always long been an excellent source of medicines, with many medications commonly available are derived from them explicitly or indirectly. In vitro cell-free α-amylase and α-glucosidase inhibition assays in a concentration range of 25 to 400 μg/mL were performed to study the anti-diabetic potential of *Aquilegia pubiflora* leaf extracts. AP_E_ has a greater efficiency against α-glucosidase (47.23 ± 0.41) and α-amylase (55.38 ± 0.73) inhibition compared to AP_DW_ (α-glucosidase: 34.14 ± 0.44 inhibition and α-amylase: 37.43 ± 0.65 inhibition) and AP_M_ (α-glucosidase: 24.63 ± 0.32 inhibition and α-amylase: 29.31 ± 0.48 inhibition) Table 4. For each experiment, acarbose (10 μM), the most commonly used therapeutic inhibitor, was used as a positive control. Our results are in harmony with previous reports [78, 79].

**Table 4:** α-glucosidase and α-amylase inhibitory potential of different plant extracts

Enzymes	Sample	Concentration				
		25 μg/mL	50 μg/mL	100 μg/mL	200 μg/mL	400 μg/mL
		% inhibition				
α-amylase	AP_DW_	17.83 ± 0.19**	26.22 ± 0.34**	29.05 ± 0.33**	32.03 ± 0.66**	37.43 ± 0.65**
	AP_M_	13.39 ± 0.17***	17.61 ± 0.24***	17.93 ± 0.31***	23.81 ± 0.39***	29.31 ± 0.48***
	AP_E_	22.82 ± 0.21*	31.08 ± 0.28*	37.83 ± 0.29*	51.26 ± 0.82*	55.38 ± 0.73*
	AP_EA_	12.37 ± 0.18***	17.92 ± 0.19***	21.28 ± 0.22***	22.71 ± 0.30***	27.23 ± 0.41***
	+ control	27.78 ± 0.66	44.87 ± 0.93	59.43 ± 1.09	73.20 ± 1.51	89.40 ± 1.24
α-glucocidase	AP_DW_	14.94 ± 0.21*	20.61 ± 0.19*	22.91 ± 0.19**	30.51 ± 0.44*	34.14 ± 0.44**
	AP_M_	11.93 ± 0.20***	16.72 ± 0.17**	20.17 ± 0.15**	20.52 ± 0.29**	24.63 ± 0.32***
	AP_E_	16.77 ± 0.34*	22.82 ± 0.21*	30.19 ± 0.43*	33.61 ± 0.31*	47.23 ± 0.41*
	AP_EA_	9.06 ± 0.11***	14.98 ± 0.18**	16.28 ± 0.21***	20.75 ± 0.27**	23.71 ± 0.31***
	+ control	27.32 ± 0.73	33.4 ± 1.27	53.45 ± 1.11	65.98 ± 1.67	84.34 ± 1.92

Star *–*** represent; *** highly significant, ** slightly significant and * non-significant difference from the control at *P* < 0.05 by one-way ANOVA in the column. Values are mean ± SD of triplicate. Where AP_DW_, AP_M_, AP_E_ and AP_EA_ stands for Distilled water, Methanol, Ethanol and Ethyl acetate extracts, respectively

### Antioxidant assay

The changes occurred in plant metabolic pathways are attributed to environmental stresses that results in production of reactive oxygen species (ROS), that can destroy membrane lipids, DNA, proteins and plant cells [80]. Plants produce numerous metabolic compounds such as flavonoids, terpenoids and oxidative stress response phenolics that act as protective mechanism against these stresses [81, 82]. This paper investigated the antioxidant ability of plant extracts by using four antioxidant assays i.e., TAC (total antioxidant capacity is based on the conversion of Mo (VI) to Mo (V) by the test material), FRAP (ferric reducing activity) assay (ET-based antioxidant activity), ABTS (2,2-azinobis-3-ethylbenzthiazoline-6-sulphonic acid) assay and TRP (total reducing power) assay as exhibited in (Table 5). Value for TAC and TRP were measured in terms of ascorbic acid equivalents of the test sample (μg AAE/mg), whereas the FRAP and ABTS activities were indicated as TEAC (trolox C equivalent antioxidant capacity, μM). The highest TAC (111.6 ± 2.9% μgAAE/mg) was shown by AP_M_ followed by AP_EA_ (97.4 ± 1.3 μgAAE/mg) at 400 μg/mL. The ABTS of AP_E_ was (277.5 ± 2.9 μgAAE/mg) followed by AP_DW_ (213.7 ± 3.1 μgAAE/mg). Similarly, the TRP was measured, where the highest value of (89.2 ± 2.4 μgAAE/mg) was shown by AP_E_ followed by the AP_DW_ (86.3 ± 2.1 μgAAE/mg). Also, the FRAP activity showed the same trend where the AP_E_ has the highest value of 289.9 ± 2.9 μM, followed by AP_EA_ 245.1 ± 3.8 μM, AP_DW_ 221.4 ± 4.1 μM and AP_M_ 198.6 ± 3.3 μM, respectively. The antioxidant properties exhibited by leaf extracts may be correlated with the presence of secondary metabolites in plants extracts. Our results match with previous reports [83–85].

**Table 5:** Showing antioxidant potential of *Aquilegia pubiflora* extracts

Concentration 200 μg/mL				
Samples Extracts	TAC (μgAAE/mg)	TRP (μgAAE/mg)	ABTS (TEAC)	FRAP (% FRSA)
AP_DW_	86.3 ± 0.9	86.3 ± 2.1	213.7 ± 3.1	221.4 ± 1.38
AP_M_	111.6 ± 2.9	76.7 ± 0.9	211.3 ± 1.9	198.6 ± 1.2
AP_E_	92.6 ± 1.8	89.2 ± 2.4	277.5 ± 2.9	289.9 ± 1.74
AP_EA_	97.4 ± 1.3	72.8 ± 0.9	188.5 ± 2.2	245.1 ± 0.97

Values are mean ± SD of triplicate. Where AP_DW_, AP_M_, AP_E_ and AP_EA_ stands for Distilled water, Methanol, Ethanol and Ethyl acetate extracts, respectively

### In vitro AChE and BChE inhibition assays

Alzheimer’s disease (AD) is a progressive neurodegenerative disease contributing to 60–80% of dementia cases worldwide. The disease is characterized by gradual decline in cognitive abilities such as memory, executive and visual spatial functioning, personality and language [86]. The prevalence rate of the disease is alarming and in United States alone a person develops AD every 65 s [87]. Current treatments available for AD includes cholinesterase inhibitors for patients with any stage of AD. Diverse synthetic and natural substances have been reported for the effective inhibition of cholinesterase enzymes. The enzyme functions by catalyzing the hydrolysis of acetyl choline (neurotransmitter) into choline and acetic acid in the synapsis or neuro-muscular junctions in the tissues. The decreased levels of acetyl choline results in the progression of AD. In the study, different concentrations of the plant extracts were tested for inhibition response of two cholinesterase enzymes i.e. Acetylcholinesterase (AChE) and butrylcholineterase (BChE) [88]. Interestingly, the inhibition response obtained for both esterases was dose dependent. AP_E_ was most active at 400 μg/mL resulted in 81.5 ± 1.6% inhibition of AChE and 83.9 ± 1.4% for BChE, followed by AP_DW_ which resulted in 76.3 ± 1.1% for AChE and 77.4 ± 0.99% for BChE, respectively. While lower inhibition response of AChE 27.6 ± 0.31% and BChE 31.1 ± 0.34% for AP_EA_ was observed at 25 μg/mL. Overall, all types of tested leaf extracts were found to be highly active against both the enzymes as indicated by their IC50 values for AChE and BChE in Table 6. Our results are matching with previous studies [89, 90].

**Table 6:** AChE and BChE inhibitory potential of plant extracts

Enzymes	Sample		Concentration					
		25 μg/mL	50 μg/mL	100 μg/mL	200 μg/mL	400 μg/mL	+ control	IC50
AChE	AP_DW_	31.7 ± 0.70***	39.2 ± 0.66***	47.5 ± 0.80***	62.7 ± 1.2*	76.3 ± 1.1*	91.2 ± 2.1	193
	AP_M_	34.2 ± 0.81***	44.6 ± 1.4**	51.7 ± 0.86*	59.1 ± 0.92*	68.5 ± 1.2*	87.1 ± 2.2	98
	AP_E_	27.8 ± 0.69***	34.9 ± 0.61**	57.4 ± 0.92*	68.3 ± 1.2*	81.5 ± 1.6*	88.9 ± 1.9	91
	AP_EA_	27.6 ± 0.31***	38.7 ± 0.50**	45.3 ± 0.73**	57.4 ± 0.81**	71.3 ± 0.81*	84.2 ± 1.7	163
BChE	AP_DW_	34.7 ± 0.93***	42.2 ± 0.62**	49.7 ± 1.2**	66 ± 0.91*	77.4 ± 0.99*	91.6 ± 1.9	154
	AP_M_	31.9 ± 0.60***	36.5 ± 0.48***	42.7 ± 0.72**	61.1 ± 0.79*	72.1 ± 1.3*	90.3 ± 1.9	121
	AP_E_	38.2 ± 0.74***	41.9 ± 0.59**	54.2 ± 1.4**	71.4 ± 0.93*	83.9 ± 1.4*	92.1 ± 1.8	84
	AP_EA_	31.1 ± 0.34**	43.1 ± 0.75**	57.3 ± 0.88*	61.6 ± 0.71*	67.1 ± 0.73*	87.3 ± 1.7	95

Star *–*** represent; *** highly significant, ** slightly significant and * non-significant difference from the control at *P* < 0.05 by one-way ANOVA in the column. Values are mean ± SD of triplicate. Where AP_DW_, AP_M_, AP_E_ and AP_EA_ stands for Distilled water, Methanol, Ethanol and Ethyl acetate extracts, respectively

### Anti-inflammatory assay

**Fig. 3:**
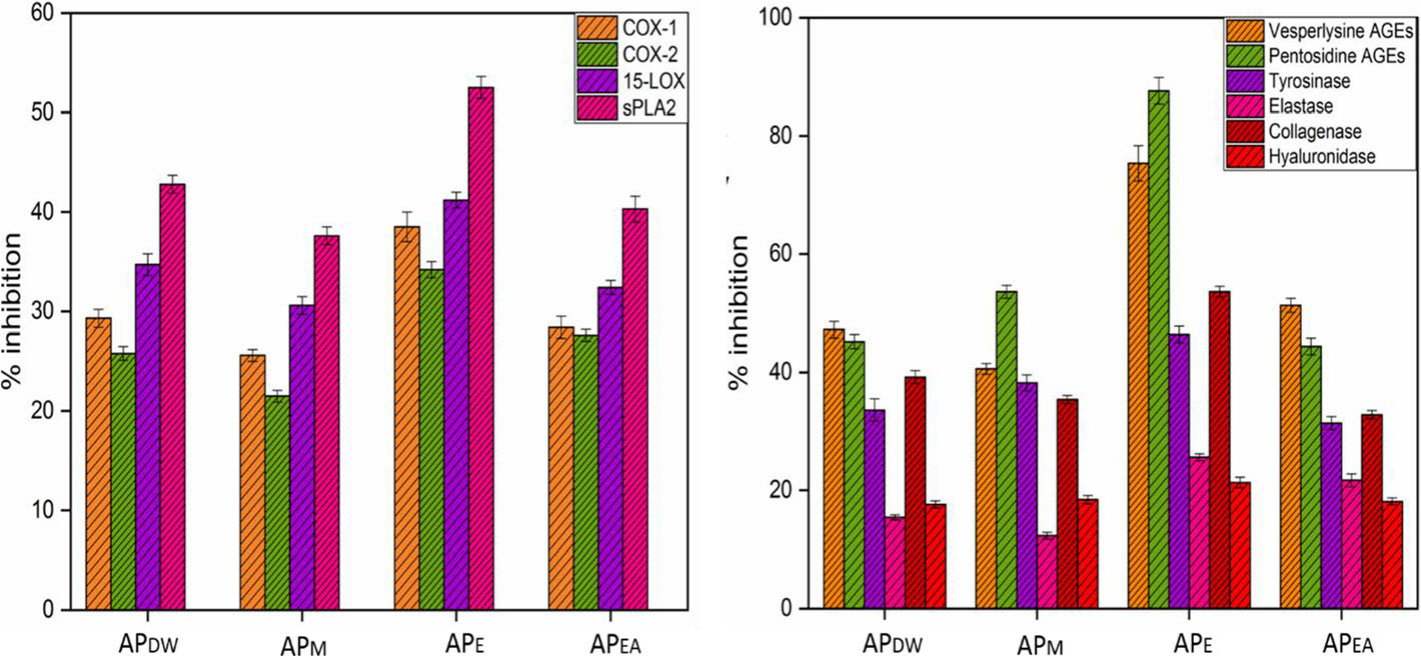
**a** Anti-inflammatory potential of medicinally important *Aquilegia pubiflora* extracts, **b** Anti-aging potential of *Aquilegia pubiflora* leaf extracts. Values are mean ± SD of triplicate. Where AP_DW_, AP_M_, AP_E_ and AP_EA_ stands for Distilled water, Methanol, Ethanol and Ethyl acetate extracts, respectively

The local vascularized or inflammatory reaction to toxins and irritants is known as inflammation. The anti-inflammatory activity of *Aquilegia pubiflora* is focused on its conventional usages [34–37]. Phenylpropanoids are one of the essential groups of plant secondary metabolites which have potential to suppress main enzymes involved in the cycle of inflammation and hence, attributing anti-inflammatory properties to the plant extracts, [91]. Anti-inflammatory activity can be performed via different pathways, including inhibition of cyclooxygenases (COX-1 and COX-2), phospholipase A2 (sPLA2), and lipoxygenase (15-LOX, enzyme-generating eicosanoids), all results in reduction in level of leukotrienes and prostanoids [92]. Specific in vitro assays such as COX-2, COX-1, sPLA2, and 15-LOX were conducted to verify the anti-inflammatory function of the test samples. The highest anti-inflammatory activity among all samples was shown by AP_E_ (52.5 ± 1.1) against sPLA2, (41.2 ± 0.8) against 15-LOX, followed by (38.5 ± 1.5) and (32.4 ± 0.8) against COX-1 and COX-2, respectively. The percent inhibition of other tested samples is shown in the Fig. 3a. Previous studies have shown that the enhanced anti-inflammatory activity is due to the phenols and flavonoids content present in *Aquilegia pubiflora* [93]. The enhanced anti-inflammatory activity of some medicinal plants has been reported in previous studies [94]. The plant phytochemicals are solely responsible for inhibiting enzymes that cause body inflammation [95]

### Anti-aging assay

This assay involved the screening of *Aquilegia pubiflora* plant extracts for their anti-aging potential. The test samples at a fixed concentration was used to evaluate their in vitro potency to inhibit enzymes such as tyrosinase, elastase, collagenase, hyaluronidase and AGEs (Fig. 3b). Collagenase, hyaluronidase and elastase like enzymes are responsible for the degradation of extracellular matrix components in the dermis. These enzymes cause skin alterations, which include deep wrinkles, skin tonus, and resilience losses [96–98]. Tyrosinase dysfunctions induce aging phenomenon and is the main causal agent of malignant melanoma and freckles or melissa like pigmentary disorders [99]. It has been reported that oxidative stress leads to provoke advanced glycation and produces (AGEs) end products which are directly associated with aging and age-related diseases [100, 101]. Such compounds, which have the potency to deter these enzymatic activities or pathways have been found to be attractive and efficient in cosmetics research. Several studies suggest that SIRT-1 (a class III deacetylase) and radical theory of aging have emerged as unique and potent agents for longevity and controlling oxidative stress effects. These agents mainly stimulate antioxidant response through FOXOs and p53 pathways [102, 103]. In our recent study, we have used *Aquilegia pubiflora* extracts, having important phytochemicals with anti-aging properties that can be exploited as anti-aging agents. We have detected strong inhibitory actions of AP_E_ toward pentosidine AGEs (up to 87.6 ± 2.26), followed by visperlysine AGEs (up to 75.4 ± 2.99) and (up to 53.6 ± 0.9) against collagenase. AP_DW_ showed intermediate inhibitory effect toward pentosidine AGEs (45.2 ± 1.2) and tyrosinase (33.6 ± 1.9). Inhibitory effects observed for elastases were lowest marked up to (25.6 ± 0.6) for AP_E,_ (21.7 ± 1.1) and (15.4 ± 0.4) for AP_EA_ and AP_DW_ respectively. From the above results, it has been elucidated that *Aquilegia pubiflora* has strong inhibitory potential against pentosidine and visperlysine AGEs.

### Anti-leishmanial activity

Leishmaniasis, is highly neglected, non-contagious tropical and subtropical infectious disease caused by parasites largely found in *Leishmania* species. According to World Health Organization (WHO), the disease is endemic in 89 countries with annual global incidence of 1.5 to 2 million cases worldwide [104]. The disease is caused by an intracellular parasite and is transmitted to humans by sand flies (Phlebotomus and Lutzomyia) bite. Due to inappropriate vector and inefficient and unaffordable drugs the disease is at high risk of uncontrolled spreading. In our study, different types of *Aquilegia pubiflora* plant extracts ranging from 25 to 400 μg/mL were investigated against both promastigote and amastigote cultures of *L. tropica* via MTT assay as shown in Table 7.A dose-dependent cytotoxicity was observed with significant mortality rate of 52% ± 1.2 and 59% ± 1.1 for AP_E_ 47% ± 0.9 and 51% ± 0.9 for AP_M_ at 400 μg/mL for promastigote and amastigote form of the parasite, respectively. Moreover, significant LC_50_ was observed for both the patristic forms i.e., 376 μg/mL for promastigote and 344 μg/mL for amastigotes by AP_E_ and 410 μg/mL for promastigote and 381 μg/mL for amastigote by AP_M._

**Table 7:** Cytotoxic potential of *Aquilegia pubiflora* plant extracts against the promastigote and amastigote forms of *Leishmania tropica* (KWH23)

*L. tropica*	Sample		Concentration					
		25 μg/mL	50 μg/mL	100 μg/mL	200 μg/mL	400 μg/mL	+ control	IC50
Promastigote	AP_DW_	16.7 ± 0.40***	21.3 ± 0.61**	26.4 ± 0.74**	40.7 ± 0.86*	44.1 ± 1.1*	73.1 ± 1.3	443
	AP_M_	14.2 ± 0.51***	24.8 ± 0.63***	31.6 ± 0.70**	41.3 ± 0.91*	47 ± 0.9*	78.8 ± 1.7	410
	AP_E_	20 ± 0.52***	24.5 ± 0.56***	37.2 ± 0.79**	45.3 ± 0.82*	52 ± 1.2*	75.2 ± 1.2	376
	AP_EA_	17.1 ± 0.51***	28.1 ± 0.50**	35.8 ± 0.81**	40.4 ± 0.73*	46.4 ± 0.81*	75.4 ± 1.1	471
Amastigote	AP_DW_	19.4 ± 0.53***	32.8 ± 0.97**	28.9 ± 0.72***	39.6 ± 0.61*	47.4 ± 0.82*	78.7 ± 1.6	464
	AP_M_	19.9 ± 0.63***	26.9 ± 0.41***	32.5 ± 0.86**	41.1 ± 0.69*	51 ± 0.9*	78.1 ± 1.6	381
	AP_E_	20.1 ± 0.64***	31.1 ± 0.69***	44.6 ± 1.1**	51.1 ± 0.83*	59 ± 1.1*	73.9 ± 1.5	344
	AP_EA_	21.4 ± 0.44***	33.8 ± 0.55**	37.8 ± 0.78**	42.3 ± 0.78*	49.1 ± 0.71*	74.9 ± 1.1	425

Star *–*** represent; *** highly significant, ** slightly significant and * non-significant difference from control at *P* < 0.05 by one-way ANOVA in the column. Values are mean ± SD of triplicate. Where AP_DW_, AP_M_, AP_E_ and AP_EA_ stands for Distilled water, Methanol, Ethanol and Ethyl acetate extracts, respectively

### Anti-cancer activity against HepG2 cell line

**Fig. 4:**
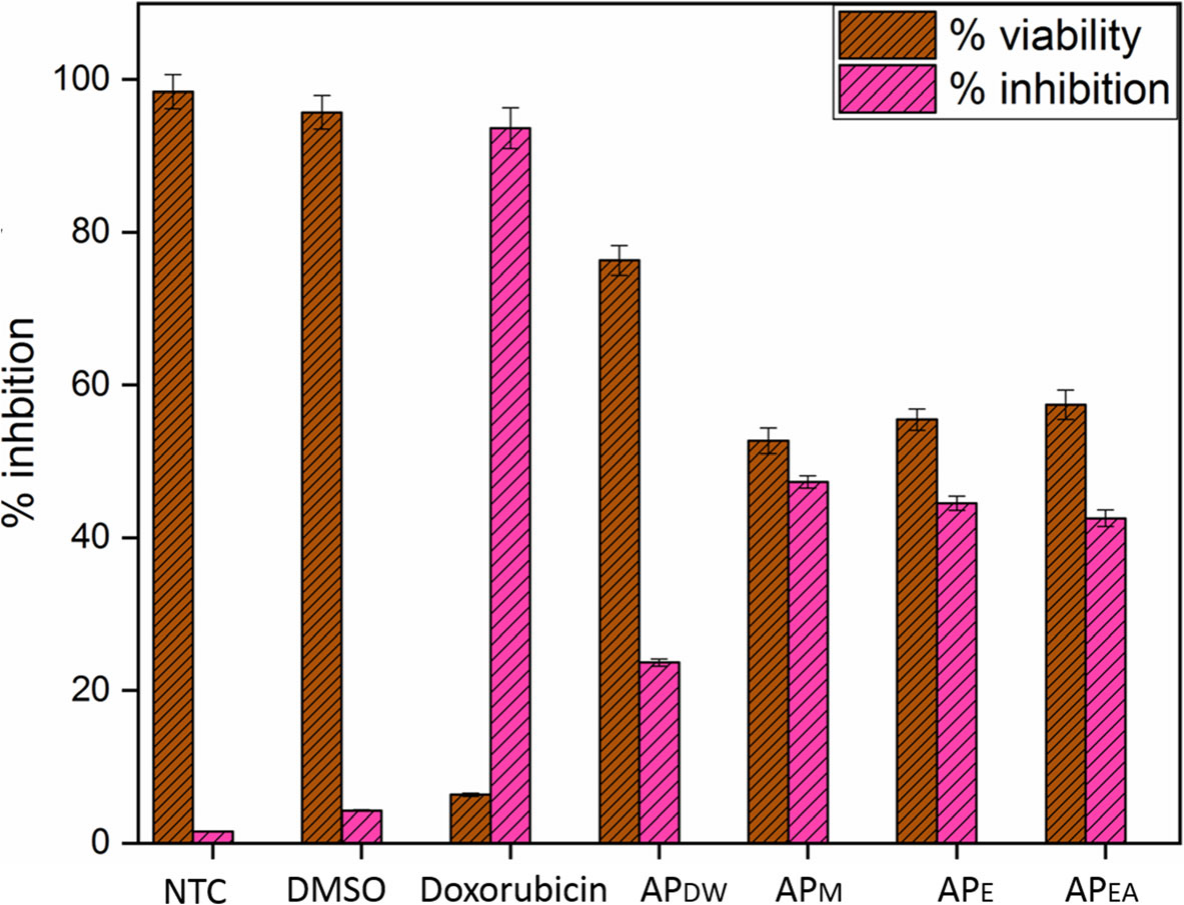
Cytotoxic potential of the *Aquilegia pubiflora* leaf extracts against the HepG2 cell line. Values are mean ± SD of triplicate. Doxorubicin was applied as positive control. Where AP_DW_, AP_M_, AP_E_, AP_EA_ and NTC stands for Distilled water, Methanol, Ethanol, Ethyl acetate extracts and Non-Treated Cells, respectively

Recently, plant-derived compounds have been regarded as a powerful and helpful alternative source for treating hepatocellular carcinoma [105]. In our study, HPLC analysis confirmed the presence of vitexins, *p*-coumaric acid and ferulic acid, phenols and flavonoids, which are physiologically important against the treatment of pathogenic illness in humans and animals [106]. The chemo-preventive and less harmful nature of these compounds with effective anticancer potential provide a research hotspot for treatment of cancer. However, due to certain issues like inadequate solubility, structural deformation and bioavailability they target a cancer site very poorly [106, 107]. We explored the potential and cytotoxicity of *Aquilegia pubiflora* extracts against human hepatocytes (HepG2 cells) as shown in Fig. 4. Tested leaf extracts showed a significant inhibition of HepG2 cells by reducing their cell viability. Doxorubicin was used as positive control and resulted in 97.38 ± 2.71% inhibition of HepG2 cells. On the other side the cytotoxicity or % cell inhibition observed for aqueous plant extracts AP_EA_, AP_E_ and AP_M_ was 50.08 ± 2.4%, 42.68 ± 2.1% and 45.68 ± 2.1%, while AP_DW_ showed % cell inhibition of 23.68 ± 2.1%, respectively. These results indicated that AP_EA_, AP_E_ and AP_M_ extracts has successfully reduced the viability of HepG2 cells above 40%, hence, exhibiting a good anti-cancer potential. The cytotoxic effects of these extracts may involve three key mechanisms, including their dissolution into functional entities, formation of reactive oxygen species (ROS) and DNA damage [108–110]. Moreover, physical properties, surface chemistry and dose dictates the overall uptake, elimination and anti-tumor properties of the test samples [110]. However, most of the available data about the in vitro anti-hepatocarcinoma activity are related to nature and presence of chemicals in plant extracts. Our findings thus augment and support the previously reported studies. The significant antitumor activity against HepG2 cell line may suggests an exciting potential of *Aquilegia pubiflora* and their extracts mediated oxide nanoparticles as promising anticancer agents.

## Discussion

Himalayan Columbine (*Aquilegia pubiflora* Wall. Ex Royle) is a medicinal plant that has been used for decades to cure skin burns, jaundice, hepatitis, wound healing, cardiovascular and circulation problems [111]. The purpose of this study was to investigate the phytochemical constituents and further evaluate the antimicrobial, anti-parasitic, anti-Alzheimer, and cytotoxic properties of the *Aquilegia pubiflora* extracts (AP_DW_, AP_M_, APE, and AP_EA_). Genus Aquilegia belongs to the *Ranunculaceae* family, which has more than 60 species of plants that are used primarily in South Asia and worldwide for many medicinal purposes. These plants have abundant phytochemicals with efficient therapeutic properties [31–33]. Leaf extracts from *Aquilegia publiflora* were made using four different solvents with varied polarity. High-performance liquid chromatography (HPLC) was used to detect and quantify the particular phytochemicals contained in these extracts. HPLC results indicated a higher number of flavonoids and derivatives of hydroxycinnamic acid. Among the detected flavonoids orientin was observed to be present in a higher concentration for AP_EA_ and AP_E_ (171 ± 2.4 μg/g DW and 983 μg/g DW respectively) while chlorogenic acid was found to be abundant hydroxycinnamic acid for both AP_EA_ and AP_E_ (1.15 ± 0.08 μg/g DW and 1.70 μg/g DW respectively). Our results are in accordance with previous reports [67, 111].

Antibiotic resistance is a severe problem that affects healthcare systems in both developing and developed countries across the world. The emergence and spread of multidrug-resistant diseases has had a significant impact on conventional antibiotic therapies [55]. As a result, in recent years, the search for novel sources of antimicrobial drugs has intensified in order to tackle resistant pathogenic diseases. Medicinal plants with antimicrobial potential have been extensively studied in this context, as they include a variety of bioactive chemicals with well-established therapeutic characteristics [56–58]. The research demonstrated that all plant extracts used had varied degrees of antimicrobial activity against all microorganisms tested at different concentrations. All the tested strains were susceptible to all extracts with *K. pneumonia* is found to be most susceptible. Inhibition zones measured at 5 mg/mL for AP_DW_, AP_M_, AP_E_ and AP_EA_ were 11.2 ± 0.47, 13.9 ± 0.33, 12.7 ± 0.41, and 13.5 ± 0.62 for *Klebsiella pneumoniae,* respectively. Our findings was close to that of previous studies that documented antibacterial activity of methanolic extract of *O. corniculata* [60]. The presence of antifungal toxicants in plant extracts may account for their antifungal effectiveness. Several authors have also observed fungicidal activity in a wide range of species. Antifungal chemicals are found in higher plants and are thought to be effective in avoiding plant infections [62]. Five pathogenic fungal strains (*A. fumigatus, A. flavus, M. racemosus, F. solani and A. niger*) were tested against *Aquilegia pubiflora* leaf extracts using the well diffusion method at concentrations in the range of 0.5 mg/mL to 5 mg/mL. The results clearly demonstrate that *A. niger* was the most susceptible strain in case of AP_DW_ with the highest zone of inhibition 14.3 ± 0.32, 13.2 ± 0.41 in case of AP_M_, 13.7 ± 0.39 for AP_E_ while 15.4 ± 0.43 zone of inhibition was recorded in case of AP_EA_ at 5 mg/mL, respectively. Previously, plant extracts from *Ageratum conyzoides* were found to have the same antifungal activity against the tested fungal specie [63, 112].

Plants have always long been an excellent source of medicines, with many medications commonly available are derived from them explicitly or indirectly. Different concentrations of the plant extracts were tested for inhibition response against α-glucosidase, α-amylase, acetylcholinesterase (AChE) and butrylcholineterase (BChE). Interestingly, the inhibition response obtained for both esterases was dose dependent. AP_E_ was most active at 400 μg/mL resulted in 81.5 ± 1.6% inhibition of AChE and 83.9 ± 1.4% for BChE, followed by AP_DW_ which resulted in 76.3 ± 1.1% for AChE and 77.4 ± 0.99% for BChE, respectively. While lower inhibition response of AChE 27.6 ± 0.31% and BChE 31.1 ± 0.34% for AP_EA_ was observed at 25 μg/mL. Overall, all types of tested leaf extracts were found to be highly active against both the enzymes as indicated by their IC50 values for AChE and BChE. Our results are matching with previous studies [89, 90]. In vitro cell-free α-amylase and α-glucosidase inhibition assays in a concentration range of 25 to 400 μg/mL were performed to study the anti-diabetic potential of *Aquilegia pubiflora* leaf extracts. AP_E_ has a greater efficiency against α-glucosidase (47.23 ± 0.41) and α-amylase (55.38 ± 0.73) inhibition compared to AP_DW_ (α-glucosidase: 34.14 ± 0.44 inhibition and α-amylase: 37.43 ± 0.65 inhibition) and AP_M_ (α-glucosidase: 24.63 ± 0.32 inhibition and α-amylase: 29.31 ± 0.48 inhibition). Our results are in harmony with previous reports [78, 79].

In our recent study, we have used *Aquilegia pubiflora* extracts, having important phytochemicals with anti-inflammatory and anti-aging properties that can be exploited for the production of cosmetics. Anti-inflammatory activity was performed via different pathways, including inhibition of cyclooxygenases (COX-1 and COX-2), phospholipase A2 (sPLA2), and lipoxygenase (15-LOX, enzyme-generating eicosanoids), all results in reduction in level of leukotrienes and prostanoids [92]. Specific in vitro assays such as COX-2, COX-1, sPLA2, and 15-LOX were conducted to verify the anti-inflammatory function of the test samples. The highest anti-inflammatory activity among all samples was shown by AP_E_ (52.5 ± 1.1) against sPLA2, (41.2 ± 0.8) against 15-LOX, followed by (38.5 ± 1.5) and (32.4 ± 0.8) against COX-1 and COX-2, respectively. Previous studies have shown that the enhanced anti-inflammatory activity is due to the phenols and flavonoids content present in *Aquilegia pubiflora* [93]. We have detected strong inhibitory actions of AP_E_ toward pentosidine AGEs (up to 87.6 ± 2.26), followed by visperlysine AGEs (up to 75.4 ± 2.99) and (up to 53.6 ± 0.9) against collagenase. AP_DW_ showed intermediate inhibitory effect toward pentosidine AGEs (45.2 ± 1.2) and tyrosinase (33.6 ± 1.9). Inhibitory effects observed for elastases were lowest marked up to (25.6 ± 0.6) for AP_E,_ (21.7 ± 1.1) and (15.4 ± 0.4) for AP_EA_ and AP_DW_ respectively. From the above results, it has been elucidated that *Aquilegia pubiflora* has strong inhibitory potential against pentosidine and visperlysine AGEs.

Protein kinase enzymes phosphorylate serine-threonine and tyrosine amino acid residues that have a key function in cellular proliferation, differentiation and apoptosis [68]. Deregulated phosphorylation by protein kinase can lead to tumor growth and entities with capability to inhibit these enzymes, are important area in anticancer research [69]. Streptomyces 85E strain was used to check the protein kinase inhibition potential of *Aquilegia pubiflora* extracts. Comparatively, AP_E_ showed the largest bald zone 14.6 ± 0.69 than AP_M_ 14.1 ± 0.35, AP_DW_ 13.8 ± 0.31 and AP_EA_ 13.2 ± 0.29 at 5 mg/mL. Lowest 5.3 ± 0.21 zone appeared in case of AP_DW_ 0.5 mg/mL representing least potential against Streptomyces hyphae growth. All the tested plant extracts inhibited Streptomyces strain at concentration-dependent manner as indicated in Fig. 2a. Overall, results showed that the entire test samples acquire vital metabolites responsible for anti-cancerous potentials in *Aquilegia pubiflora*. Our results are strongly supported by a previous report, which was conducted on the evaluation of hyphae formation inhibition in *Streptomyces* 85E. After preliminary evaluation of test extracts against protein kinase enzyme we explored the inhibition potential of *Aquilegia pubiflora* extracts against human hepatocytes (HepG2 cells). Tested leaf extracts showed a significant inhibition of HepG2 cells by reducing their cell viability. Doxorubicin was used as positive control and resulted in 97.38 ± 2.71% inhibition of HepG2 cells. On the other side the cytotoxicity or % cell inhibition observed for aqueous plant extracts AP_EA_, AP_E_ and AP_M_ was 50.08 ± 2.4%, 42.68 ± 2.1% and 45.68 ± 2.1%, while AP_DW_ showed % cell inhibition of 23.68 ± 2.1%, respectively. These results indicated that AP_EA_, AP_E_ and AP_M_ extracts has successfully reduced the viability of HepG2 cells above 40%, hence, exhibiting a good anti-cancer potential. The cytotoxic effects of these extracts may involve three key mechanisms, including their dissolution into functional entities, formation of reactive oxygen species (ROS) and DNA damage [108–110].

## Conclusion

The study findings indicated that the solvent form and polarity have an impact on extraction effectiveness, biological performance, and the quality of the pharmacological reaction. If a multi range polarity based solvent method is used in the preliminary screening stages, followed by extract optimization and bioactivity guided isolation of potentially active compounds, better results can be obtained. Different plant extracts tested have demonstrated excellent antibacterial and antifungal activities, especially against *K. pneumonia* and *A. niger*. Similarly, both amastigote and promastigote variants of the parasite *Leishmania tropica* were found to be particularly susceptible to test samples. We discovered that these plant extracts can be used to treat hepatocarcinoma because they showed significant cytotoxicity against HepG2 cells. Furthermore, substantial inhibition activity against α-amylase, α-glucosidase, Acetylcholinesterase (AChE), and Butrylcholinesterase (BChE) was observed, paying the way for its use as anti-diabetic and anti-Alzheimer’s therapeutics. Pentosidine-Like AGEs were shown to have a high benefit and anti-inflammatory capacity. The findings from the abovementioned biological activities suggested that *Aquilegia pubiflora* leaf extract could be a suitable candidate for various biomedical applications.

## Data Availability

The datasets used and analyzed during the current research work are available from the corresponding author on reasonable request.

## Abbreviations

TAC: Total antioxidant capacity
AD: Alzheimer’s disease
TRP: Total reducing power
FRSA: Free radical scavenging assay
AChE: Acetylcholinesterase
BChE: Butyrylcholinesterase
DMSO: Dimethyl sulfoxide
